# Procognitive impact of ciproxifan (a histaminergic H_3_ receptor antagonist) on contextual memory retrieval after acute stress

**DOI:** 10.1111/cns.13113

**Published:** 2019-05-15

**Authors:** Frédéric Chauveau, Elodie De Job, Betty Poly‐Thomasson, Raphaël Cavroy, Julien Thomasson, Dominique Fromage, Daniel Beracochea

**Affiliations:** ^1^ IRBA (Institut de Recherche Biomédicale des Armées) BP73 Bretigny‐sur‐Orge Cedex France; ^2^ INCIA (Institut de Neurosciences Cognitives et Intégratives d’Aquitaine), UMR CNRS 5287 Université de Bordeaux Pessac France

**Keywords:** H3 receptor antagonist, procognitive compounds, stress

## Abstract

**Aim:**

Although cognitive deficits commonly co‐occur with stress‐related emotional disorders, effect of procognitive drugs such as histaminergic H_3_ receptor antagonists are scarcely studied on memory retrieval in stress condition.

**Methods:**

*Experiment 1*. Memory of two successive spatial discriminations (D1 then D2) 24 hours after learning was studied in a four‐hole board in mice. H3 receptor antagonist ciproxifan (*ip* 3 mg/kg) and acute stress (three electric footshocks; 0.9 mA; 15 ms) were administered 30 and 15 minutes respectively before memory retrieval test. Fos immunostaining was performed to evaluate the neural activity of several brain areas. *Experiment 2*. Effects of ciproxifan and acute stress were evaluated on anxiety‐like behavior in the elevated plus maze and glucocorticoid activity using plasma corticosterone assay.

**Results:**

*Experiment 1*. Ciproxifan increased memory retrieval of D2 in nonstress condition and of D1 in stress one. Ciproxifan mitigated the stress‐induced increase of Fos expression in the prelimbic and infralimbic cortex, the central and basolateral amygdala and the CA1 of dorsal hippocampus. *Experiment 2*. Ciproxifan dampened the stress‐induced anxiety‐like behavior and plasma corticosterone increase.

**Conclusion:**

Ciproxifan improved contextual memory retrieval both in stress and nonstress conditions without exacerbating behavioral and endocrine responses to stress. Overall, these data suggest potential usefulness of H_3_ receptor antagonists as cognitive enhancer both in nonstress and stress conditions.

## 1 INTRODUCTION

The histamine H_3_ receptor is a central inhibitory autoreceptor located on histaminergic nerve terminals that are found mainly on cholinergic and dopaminergic neurons. H3 receptor activation resulted in a reduction of the release of histamine in the brain, whereas its inhibition (by inverse agonist or antagonist) increased the release of histamine^1^, ^2^, ^3^. Ciproxifan (cyclopropyl 4‐(3‐(1H‐imidazol‐4‐yl)propyloxy) phenyl ketone) is an extremely potent histamine H_3_ receptor (H_3_R) inverse agonist/antagonist which enhanced the release of histamine and increases sustained attention and alertness states.^4^, ^5^, ^6^ Both thioperamide (a potent HRH3 antagonist) and ciproxifan enhance working memory^4^, ^7^, ^8^, ^9^, ^10^ and long‐term memory^11^, ^12^, ^13^, ^14^, ^15^, ^16^, ^17^ and counteract scopolamine‐induced amnesia.^11^, ^12^, ^18^, ^19^, ^20^, ^21^

The therapeutic use of procognitive compounds might be integrated into a stressful context. Indeed, patients suffering cognitive deficits often show anxiety disorders^22^ and stress impairs hippocampus‐dependent memory retrieval.^23^ The deleterious effect of stress on cognitive functions is observed in stress‐related disorders such as anxiety and depression.^24^, ^25^, ^26^ In such context, histaminergic system is a relevant target because histamine is an indicator of stress response^27^, ^28^, ^29^, ^30^, ^31^, ^32^ since stress is a potent activator of histamine neurons in the tuberomammillary nucleus of the hypothalamus.^33^ A consensus view is that histamine antagonists have more impact in tasks having an anxiety component.^34^ For example, it has been found that ciproxifan prevented the deleterious effects of chronic stress exposure in spatial memory.^35^ In contrast, central administration of histamine can also increase plasma corticosterone via its action on hypothalamic neuropeptides^36^ and promotes anxiety‐like behavior.^37^, ^38^, ^39^, ^40^ Research efforts are now needed to determine emotional impact of H_3_R antagonists at procognitive dose.

Although numerous studies concerned procognitive action of 3 mg/kg ciproxifan in control (nonstressed) conditions,^4^, ^8^, ^9^, ^10^, ^11^ its cognitive action in hippocampus‐dependent memory in stress condition has not been yet studied and more particularly at the retrieval phase.^41^ To that aim, we investigated the effects of an acute stress (three electric footshocks 0.9 mA) on memory in a contextual serial spatial discrimination task (CSD) where stress is not directly associated with the memory task. In the CSD task, mice learned two successive discriminations and are tested 24 hours later for memory of the first or second discrimination using distinct internal context in mice.^42^ We previously showed that memory of the first discrimination involved the dorsal hippocampus in nonstress condition, whereas memory of the second discrimination involved the prefrontal cortex and the amygdala in stress condition.^43^, ^44^ Using this behavioral model, we investigated in a first experiment the effects of pre‐test injection of 3 mg/kg ciproxifan on memory of D1 and D2 in nonstress or stress conditions. Fos immunohistochemistry has been found to be a powerful tool for identifying the modifications of neural activity in brain areas particularly after stress. For example, the number of immunostained cells was increased in the amygdala of animals submitted to stressful situations.^45^, ^46^ Therefore, in a first experiment, the impact of ciproxifan on Fos expression was performed in brain areas involved in the CSD behavioral task such as the prefrontal cortex (PrL for prelimbic and Il for infralimbic cortex), the dorsal hippocampus (CA1, CA3 for Cornu Ammonis areas 1 and 3 and DG for dentate gyrus) and lateral (LA), basolateral (BLA) and central (CeA) nucleus of amygdala. A second experiment was designed to study the effect of ciproxifan 3 mg/kg on emotional reactivity in the elevated plus maze (EPM) behavior and plasma corticosterone levels.^47^ This second experiment was designed to determine if the 3 mg/kg procognitive dose of ciproxifan could be dissociated from its emotional impact.

## 2 MATERIAL AND METHODS

### 2.1 Animals

144 subjects were 3‐month‐old male mice of the C57Bl/6J inbred strain obtained from Charles River and assigned to Experiment 1 (eight groups of 13; n = 104) and Experiment 2 (four groups; n = 40). All mice were maintained in a ventilated colony room at 22 ± 1°C, under a 12:12 light‐dark cycle (lights on at 7:00 am). They were provided with food and water ad libitum. Mice were housed (5 animals/cage) during 4 weeks after arrival then were single‐housed 1 week before the beginning of each experiment. All experiments were performed in accordance with the European Communities Council Guidelines (Directive 2010/63/EU) and the local ethical committee (IMASSA#1101).

### 2.2 Procedures

#### 2.2.1 Experiment 1: effects of ciproxifan on memory and neural activities

##### Contextual serial discrimination (CSD) task

**Figure 1:**
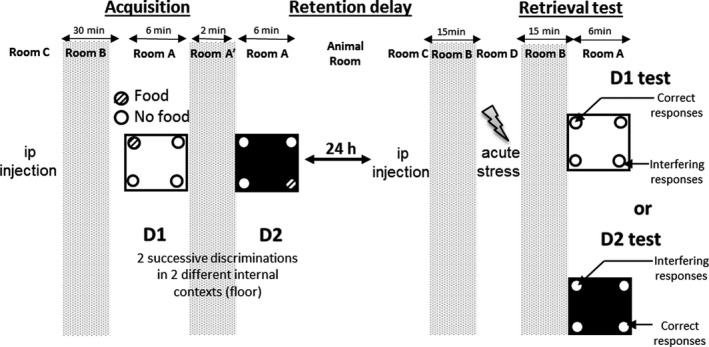
Contextual Serial spatial Discriminations (CSD) protocol. Mice were *ip* injected (vehicle solution for all groups) 30 min before acquisition phase then were exposed to the first discrimination D1 (only one hole on 4 is randomly baited by ten 20‐mg saccharose pellets) on a specific floor (white and smooth; random use for each mouse). Note that each treatment is done in a specific room. In the second discrimination (D2), the baited hole is in the opposite corner than discrimination 1 and the color and texture of the floor is changed (black and rough). On the following day, mice were randomly treated (*ip* injection and acute stress, 30 and 15 min before behavioral test respectively). During the retrieval test phase, no hole is baited and mice were randomly exposed to either D1 or D2 floor (independent groups) after treatment.

After 3 days of food restriction to maintain 90% of initial body weight, mice learn two spatial discriminations (D1 then D2) in a four‐holeboard apparatus (45 × 45 × 30 cm; Room A; Figure 1). On the floor, four holes opening on a food cup (three diameter × 2.5 cm in depth) were located 6 cm away from the sidewalls. During the acquisition session, the two serial discriminations differed by the color (black versus white) and texture (rough versus smooth) of the floor. For D1, ten 20‐mg saccharose pellets (BIOSERV, France) were available only in one randomly chosen hole. For D2, ten pellets were located in the opposite symmetrical hole. The environmental spatial cues (outside the board) were made of colored and striped paper sheets positioned at 1.00 m above the four‐hole board. These allocentric cues remained at the same place for both D1 and D2 discriminations and also for the memory retrieval test. Thus, both discriminations D1 and D2 differed only by way of the internal (floor) contextual cues.

24 hours after acquisition, memory retrieval was tested either on D1 or on D2 using the specific floor of each acquisition. Mice were allowed to freely explore the apparatus and performance was assessed by measuring the number of head‐dips in each hole during 6 minutes without any pellets in the apparatus. All mice tested in the retrieval phase were included in statistics. The following parameters were calculated: (a) % of correct responses (number of head‐dips into the hole previously baited on the same floor‐context/total number of head‐dips × 100), (b) % of spatial responses: interfering responses (number of head‐dips into the hole previously baited on the other floor‐context/total number of head‐dips × 100) + correct responses. Spatial responses refer specifically to head‐dips into the two previously baited holes, regardless of the floor used at the acquisition phase. Thus, within the framework of our analysis, spatial responses depended exclusively on knowledge of the external allocentric cues which remained stable over the learning of D1 and D2 and during the test phase. Conversely, “correct” responses emerges as an index of contextual memory which can be considered as reflecting a unique event‐related memory.^48^

##### Drug administration

All animals were injected intraperitoneally with vehicle 30 minutes before the acquisition phase (0.9% saline solution; 0.1 ml/10 g body weight; Figure 1). During the test session, the animals randomly received either the vehicle solution or ciproxifan (3 mg/kg/body weight diluted in a 0.9% saline solution) 30 minutes before behavioral assessment. The dose of ciproxifan was chosen according to previous studies.^4^, ^8^, ^9^, ^10^, ^11^

##### Stress administration

Fifteen minutes before behavioral test (CSD and EPM tests), mice were randomly chosen and placed in the stress delivery cage for 1 minute. Three consecutive inescapable electric footshocks (0.9 mA; 15 ms) were delivered every 20 seconds.

##### Immunohistochemical procedure

Ninety minutes after test, mice were killed under deep anesthesia (ketamine 200 mg/kg, xylazine 20 mg/kg, ip) and their brain removed after 4%‐paraformaldehyde perfusion. Brain slices (50 µm thickness) were incubated overnight at 4°C with a primary antibody specific of Fos protein (PC38, Calbiochem), then with a biotinyled secondary antibody (Interchim) 2 hours at room temperature and finally with the avidine‐biotine‐peroxydase complex Vectastain^®^ (Abcys). C‐Fos immunoreactivity was revealed using NovaRed^®^ peroxydase substrate kit (Vector Laboratories). The analysis was conducted in the prefrontal cortex (PrL for prelimbic and Il for infralimbic cortex), dorsal hippocampus (CA1, CA3 for Cornu Ammonis areas 1 and 3 and DG for dentate gyrus) and amygdala (LA, BLA, CeA for lateral, basolateral and central nucleus respectively) according to the mouse stereotaxic brain atlas of Paxinos and Franklin.^49^ Digital images were captured at 10× magnification using an Olympus (BX50) and analyzed by image analysis software (Icy version 1.7.3.0;icy.bioimageanalysis.org). At all stages, the experimenter was blind to the experimental groups. Slices showing clear and reliable Fos staining were pooled from animal performing either D1 or D2. Three sections from each animal were examined bilaterally, and the number of positive nuclei/mm^2^ was averaged (8‐12 animals/group; 3 slices per animal) and were expressed in mean counts ± SEM in relative variations as compared to naive controls (food deprived animal staying in animal room that were excluded from the acquisition phase).

#### 2.2.2 Experiment 2: effects of ciproxifan and stress on behavioral and endocrinal reactivity

##### General protocol

Drug injections (vehicle or ciproxifan 3 mg/kg) and stress (three footshocks 0.9 mA) were performed respectively 30 minutes and 15 minutes before EPM. Mice were randomly assigned to four experimental groups (NS Veh; NS Cipro3, Str Veh; Str Cipro3).

##### Elevated plus maze

The EPM consisted of two open‐arms (30 cm long, 7 cm wide) and two closed‐arms (side walls 24 cm high) elevated 38 cm above the ground. Light intensity was controlled before experiment (100 lux in open‐arms; <10 lux in closed‐arms). Behavior was recorded 5 minutes by a videotracking system (Viewpoint, France) allowing to measure the travelled distance and running time. Two measures of anxiety‐like reactivity were taken. The first was the distance into the open‐arms and the second was the ratio of the time spent in the open‐arms divided by the total time spent in all arms (time ratio). The exploration of open‐arms is negatively correlated to the anxiety‐like state. The exploration in closed‐arms is used as an index of locomotor activity.

##### Plasma corticosterone

At the end of the EPM task, animal were decapitated, trunk blood was immediately centrifuged at 4°C and plasma was stored at −20°C until corticosterone assay. Corticosterone concentrations were quantified using a commercially Enzyme Immunoassay kit (DetectX, Arbor Assays). The limit of detection of this assay was 1.7 µg/dL that is 4‐fold lower than the minimal value (8.05 µg/dL) obtained in the present study.

#### 2.2.3 Statistical analysis

The normality of the distribution was evaluated using Shapiro‐Wilk test. Data are displayed with bar graph representing mean ± SEM (standard error mean). One or two‐way factorial analysis of variance (ANOVA) of drug and stress factors were performed for behavioral analyses (experiment 1 and 2) and plasma corticosterone assay followed by post hoc comparisons (Scheffe multiple comparison test; two‐sided method adjustment). Insofar that stress increase Fos immunoreactivity,^50^ the interaction between drug and stress factors was not performed. Therefore, drug effect on Fos staining in nonstressed animals was analyzed independently of stressed animals by the mean of unpaired *t* test. The significance level was set at *P* < 0.05, nonsignificant results are reported as NS. Statistical analysis was performed using Statistica^®^ 7.0. software.

## 3 RESULTS

### 3.1 Experiment 1: Impact of ciproxifan on memory retrieval and neural activities in stress and nonstress conditions

#### 3.1.1 Contextual serial discrimination (CSD)

*Acquisition.* The number of animals per group is mentioned in Table 1. No significant between‐groups difference was observed both for the total number and the percentage of head‐dips in the baited hole, both for discrimination 1 and Discrimination 2) (*P* > 0.10 in all analyses).

**Table 1:** Behavioral performances during acquisition phase in CSD task

Treatment for retrieval test phase			N	Acquisition Discrimination 1		Acquisition Discrimination 2	
DRUG	STRESS	DISCRI		Total head‐dips	% baited hole visits	Total head‐dips	% baited hole visits
VEH	NS	D1	11	46.5 ± 5.9	37.9 ± 5.2	59.4 ± 6.9	57.2 ± 2.7
VEH	NS	D2	13	48.1 ± 4.2	38.5 ± 4.1	61.4 ± 6.8	55.1 ± 6.2
CIPRO3	NS	D1	13	50.8 ± 7.1	42.5 ± 6.3	65.8 ± 5.6	54.5 ± 5.1
CIPRO3	NS	D2	11	51.5 ± 4.1	44.8 ± 6.1	63.4 ± 7.1	58.4 ± 4.3
VEH	STRESS	D1	10	50.1 ± 7.7	40.3 ± 5.2	66.7 ± 5.1	56.8 ± 3.8
VEH	STRESS	D2	13	49.2 ± 8.4	39.6 ± 9.8	65.2 ± 8.1	63.6 ± 5.5
CIPRO3	STRESS	D1	12	48.9 ± 6.5	43.1 ± 7.5	62.5 ± 4.1	59.2 ± 5.1
CIPRO3	STRESS	D2	12	50.7 ± 7.2	40.9 ± 8.3	69.3 ± 6.1	60.8 ± 3.9
TOTAL MEAN				49.8 ± 7.5	41.0 ± 8.4	64.2 ± 9.7	58.2 ± 9.1

For the eight studied groups, effective (“N” column), discrimination (DISCRI), drug (VEH = vehicle; CIPRO3 = ciproxifan 3 mg/kg) and stress (stress = 3 acute electric footshocks 0.9 mA; NS = nonstress) are mentioned. Data are expressed by the mean + SEM of total head‐dips (four holes) and % visit of the baited hole (1 on 4) during 6 min of acquisition phase for Disciminitation 1 (D1) and for Discrimination 2 (D2). All mice are included in the memory retrieval test.

**Figure 2:**
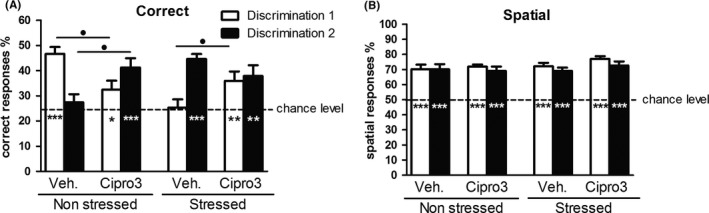
Memory retrieval performances in CSD task. Percentage of correct (A) and spatial (correct + interfering; B) responses are represented by the mean + SEM as a function of group (Veh for vehicle; Cipro3 for ciproxifan 3 mg/kg), stress and discrimination. Comparison to chance levels (25% for correct responses and 50% for spatial responses; Student *t* test): *P < 0.05, **P < 0.01, ***P < 0.001. Ciproxifan effect (Scheffe post hoc test): •*P* < 0.05, ••*P* < 0.01. For clarity, post hoc analyses of stress and discrimination effects are not mentioned. The numbers of mice per group are mentioned on Table 1 according mice included in the acquisition phase are all included in the analysis of memory retrieval test

Test phase (see Figure 2).

##### Correct responses

Percentages of correct responses were above chance level (25%) for Vehicle‐D1, Cipro‐D1 and Cipro‐D2 in nonstressed animals (*t* = 7.96, *P* < 0.0001; *t* = 2.04, *P* < 0.05; *t* = 4.5, *P* < 0.01, respectively) and for Vehicle‐D2, Cipro‐D1 and Cipro‐D2 in stressed mice (*t* = 10.01, *P* < 0.0001; *t* = 2.95, *P* < 0.01 and *t* = 2.98, *P* < 0.01 respectively). A global ANOVA revealed a significant interaction between drug, discrimination and stress factors (F(3, 87) = 20.3; *P* = 0.0001;Figure 2A).

##### Correct responses in nonstressed animals

###### Ciproxifan effect

In nonstress condition, ANOVA revealed a significant interaction between drug and discrimination (F(2, 41) = 19.70; *P* < 0.0001). Ciproxifan 3.0 mg/kg decreased the % of correct responses at D1 (32.4% ± 6.6% for ciproxifan group versus 48.5% ± 2.3% for vehicles; Scheffe post hoc test, *P* < 0.05) but increased D2 correct responses rates (41.3% ± 3.6% vs 27.6% ± 3.0%; *P* < 0.05).

###### Discrimination effect

Vehicle‐treated mice exhibited a higher % of correct responses for D1 (48.5% ± 2.3%) as compared to D2 (27.6% ± 3.0%; Scheffe post hoc test: *P* < 0.01) whereas correct responses % of ciproxifan‐treated animals were not different between both discrimination (32.4% ± 3.6% vs 41.3% ± 3.6% for D1 and D2 respectively; NS).

##### Correct responses in stressed animals

###### Ciproxifan effect

In stress condition, ANOVA also revealed a significant interaction between drug and discrimination (F(2, 46) = 5.56; *P* < 0.05): ciproxifan increased the % of correct responses at D1 (3.6% ± 3.7% for ciproxifan group versus 25.3% ± 3.3% for vehicles; Scheffe post hoc test, *P* < 0.05) but spared correct responses % for D2 (37.9% ± 4.3% vs 44.7% ± 2.0%; NS).

###### Discrimination effect

Vehicle‐treated mice exhibited a higher % of correct responses for D2 (44.7% ± 2.0%) as compared to D1 (25.3% ± 3.3%; Scheffe post hoc test: *P* < 0.01) whereas correct responses % of ciproxifan‐treated animals were not different between both discrimination (36.0% ± 3.7% vs 37.9% ± 4.3% for D1 and D2 respectively; NS).

##### Stress effect on correct responses

In vehicle group, stress differently altered correct responses rates as a function of discrimination (interaction between discrimination and stress: F(2, 38) = 54.97; *P* < 0.0001): it decreased the retrieval of D1 correct responses (from 48.5% ± 2.3% for nonstressed Veh. animals to 25.3% ± 3.3% for stressed Veh. group; *P* < 0.0001) but increased correct responses for D2 (from 27.6% ± 3.0% for NS Veh. to 44.7% ± 2.0% for Str.Veh.; *P* < 0.001). In contrast, for ciproxifan‐treated animals, stress did not alter correct responses whatever the discrimination (F(2, 49) = 0.80, NS).

##### Spatial responses

A global ANOVA revealed no significant interaction between drug, discrimination and stress factors (F(3, 87) = 0.04; NS; Figure 2B). Performance of spatial responses were all above chance level (50%; *t* = 6.76, *t* = 6.01, *t* = 15.32, *t* = 6.82, *t* = 9.16, *t* = 9.18, *t* = 14.23 and *t* = 8.69, *P* < 0.0001 for all groups, respectively, vehicle NS‐D1, vehicle NS‐D2, cipro3 NS‐D1, cipro3 NS‐D2, vehicle Str‐D1, vehicle Str‐D2,,cipro3 Str‐D1 and cipro3 Str‐D2).

#### 3.1.2 Fos expression in hippocampus, prefrontal cortex and amygdala

In all groups, CSD behavioral test increased significantly Fos immunostaining as compared to naive (nonbehaving) mice (*P* < 0.0001 in all comparisons and for all brain areas).

##### Ciproxifan effect

**Figure 3:**
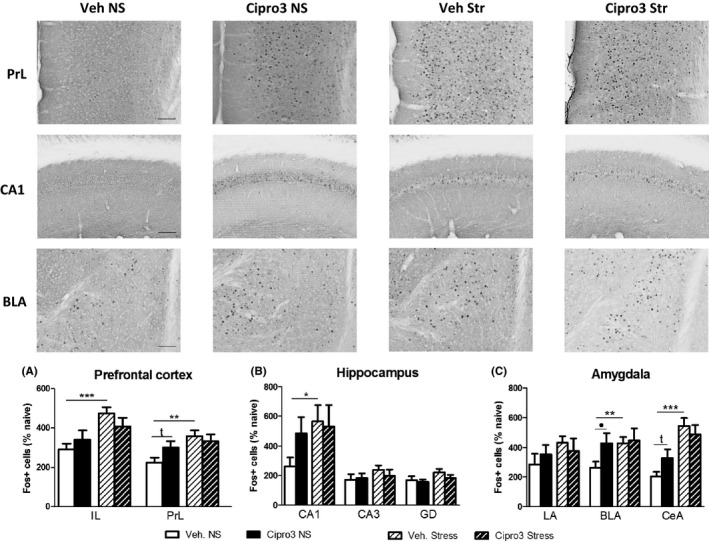
Fos protein expression induced by acute stress and ciproxifan after CSD task. *Upper part*. Representative photomicrographs of Fos immunopositive neurons in the PL, the CA1 of the hippocampus and the BLA of the amygdala (magnification ×20; scale bar: 100 µm). *Lower part*. Fos positive cell number are expressed by mean + SEM relative to naive animal level for prefrontal cortex (IL = infralimbic cortex; PrL = prelimbic cortex; A) dorsal hippocampus (CA1; CA3 for cornu ammonis; DG = dentate gyrus; B) and amygdala (LA = lateral amygdala; BLA = basolateral amygdala; CeA = central amygdala; C). Mean were calculated from three representative brain slices for vehicle (Veh) and ciproxifan 3 mg/kg (Cipro3) groups. Stress effect (unpaired *t* test): *P < 0.05, **P < 0.01, ***P < 0.001. Ciproxifan effect (unpaired *t* test): •*P* < 0.05. The numbers of mice per group are: Il (11, 8, 12, 10), PrL (11, 8, 12, 10), Hippocampus (12, 13, 15, 13), Amygdala (12, 9, 13, 11) for Vehicle Nonstress, Ciproxifan Nonstress, Vehicle Stress and Ciproxifan Stress respectively.

In nonstressed animals, ciproxifan increased Fos positive cells significantly in the BLA (ratio ×1.7; NS Veh. versus NS Cipro3, unpaired *t* test: *t* = 2.20, *P* < 0.05; Figure 3C) and with a trend in CeA (ratio: ×1.8; *t* = 1.96, *P* = 0.06) and PrL (ratio: ×1.3; *t* = 1.87, *P* = 0.07; Figure 3A). No ciproxifan effect was observed in stressed animals.

##### Stress effect

In vehicle‐treated mice, stress significantly increased Fos expression in the Il (ratio: ×1.6; NS Veh. versus Str. Veh.; *t* = 4.19, *P* < 0.001), PrL (ratio: ×1.6; unpaired *t* test, *t* = 3.52, *P* < 0.01), the CA1 (ratio: ×2.5; *t* = 2.22, *P* < 0.05), BLA (ratio: ×1.6; *t* = 2.84, *P* < 0.01) and CeA (ratio ×2.7; *t* = 5.46, *P* < 0.0001). In contrast, stress did not impair Fos expression in ciproxifan‐treated animals in any brain area.

### 3.2 Experiment 2

#### 3.2.1 Elevated plus maze

**Figure 4:**
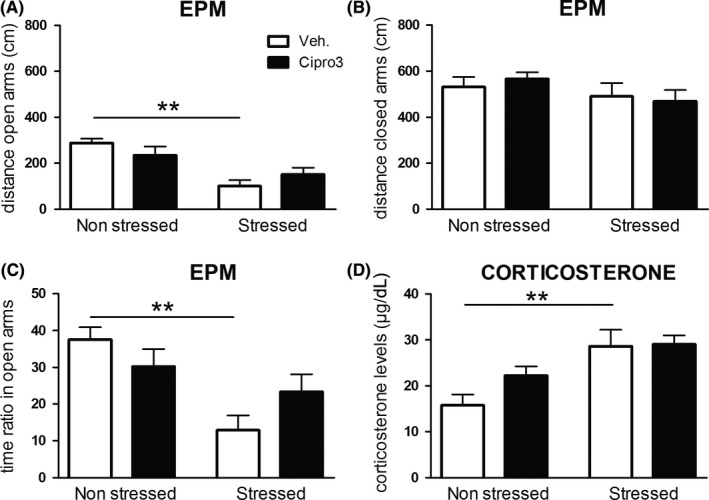
Elevated plus maze and corticosterone levels (experiment 2). Distance in open‐arms and closed‐arms are expressed in centimeters and represented by the mean + SEM for nonstressed (left bar graph) and stressed (right) animals. Ciproxifan 3 mg/kg (Cipro3; black bar graph) and vehicle (Veh; white) were *ip* administered 30 min before EPM behavior. Corticosterone levels (µg/dL) were obtained from trunk blood collected immediately after EPM behavior. Stress effect (Scheffe post hoc test): **P < 0.01. The numbers of mice per group are as follows: n = 11, 7, 14, and 8 for Vehicle Nonstress, Ciproxifan Nonstress, Vehicle Stress and Ciproxifan Stress, respectively

*Distance travelled in open‐arms* (Figure 4A). A two‐way ANOVA performed in all groups showed a nonsignificant effect of drug (F(1, 36) < 1.0), a significant effect of stress (F(1, 36) = 16.9; *P* = 0.001) and a nonsignificant drug and stress interaction (F(2, 36) = 2.5; *P* = 0.12).

##### Stress effect

No significant stress effect on open arm distance was found in ciproxifan‐treated mice (235.1 ± 37.2 cm vs 151.2 ± 29.7 cm for nonstressed and stressed ciproxifan‐treated animals respectively; NS). In contrast, stress reduced the distance travelled in open‐arms in vehicle‐treated animals (288.6 ± 18.2 cm vs 100.6 ± 25.9 cm for nonstressed and stressed vehicle‐treated animals, respectively; *P* < 0.01).

*Distance travelled in closed‐arms* (Figure 4B). A two‐way ANOVA performed in all groups showed a nonsignificant effect of drug (F(1, 36) < 1.0), of stress (F(1, 36) = 3.50; NS) neither a significant interaction between drug and stress factors (F(2, 36) = 0.41; NS).

*Time ratio in open‐arms* (Figure 4C). A two‐way ANOVA performed in all groups showed a nonsignificant effect of drug (F1, 36) = 0.1) but a significant effect of stress (F(1, 36) = 11.3; *P* = 0.01) and a trend toward significance on the interaction between factors (F(2, 36) = 3.59; *P* = 0.06).

##### Ciproxifan effect

Ciproxifan did not significantly modify the open arm time ratio both in nonstressed (30.2% ± 4.8%; vs 37.5% ± 3.7%, respectively, for NS Cipro3 and NS Veh. groups; NS) and stressed animals (23.3% ± 4.8%; vs 12.9% ± 4.0% respectively for Cipro3 and Veh. groups; NS).

##### Stress effect

No significant stress effect was found in ciproxifan‐treated mice (from 30.2% ± 4.8% to 23.3% ± 4.78% for nonstress and stress ciproxifan‐treated groups respectively; NS). In contrast in vehicles, stress decreased the percentage of time in open‐arms (from 37.5% ± 3.4% to 12.9% ± 4.0% for nonstress and stress vehicle‐treated groups respectively; *P* < 0.01).

*Ratio Entry*. A two‐way ANOVA performed in all groups showed a nonsignificant effect of drug (F(1, 36) = 0.75), a nonsignificant effect of stress (F(1, 36) = 0.58) and a nonsignificant drug and stress interaction (F(2, 36) = 0.89;data not shown).

#### 3.2.2 Plasma corticosterone levels

A two‐way ANOVA performed in all groups showed a significant effect of stress (F(1, 36) = 15.52; *P* < 0.001), but not of drug (F(1, 36) = 1.91; NS) and a nonsignificant drug and stress interaction (F(2, 36) = 1.45; NS; Figure 4D).

##### Stress effect

Stress did not significantly alter plasma corticosterone levels in ciproxifan‐treated animals (from 22.2 ± 2.0 µg/dL to 29.1 ± 1.9 µg/dL, respectively, for NS Cipro3 and Str Cipro3 groups; NS) whereas it increased levels of the vehicle group (from 15.8 ± 2.3 µg/dL to 28.6 ± 3.6 µg/dL, respectively, for NS vehicle and Str Vehicle: *P* < 0.01).

## 4 DISCUSSION

Main results are as follows. In experiment 1, ciproxifan (3 mg/kg) enhanced contextual memory retrieval both in nonstress and stress conditions. Ciproxifan increased Fos expression in the basolateral amygdala only in nonstress condition. Stress increased number of Fos positive cells in prelimbic and infralimbic cortex, hippocampus (CA1) and amygdala (basolateral and central nuclei) only in vehicle‐treated animals. In experiment 2, stress increased anxiety‐like behavior and plasma corticosterone only in vehicles showing a dampening effect of ciproxifan (3 mg/kg) both on emotional and endocrinal reactivity to stress.

### 4.1 Stress, ciproxifan, and memory retrieval

It has been reported that compounds activating histamine receptors H1 and/or H2 or increasing histamine release (via H3 histamine receptor blockade) substantially improve memory processes.^4^, ^7^, ^8^, ^9^, ^11^, ^12^, ^13^, ^14^, ^15^, ^16^ In our study, ciproxifan did not modify spatial memory performance that were already high (around 70%) in control animals, suggesting likely a ceiling effect. In contrast, a procognitive impact of ciproxifan is observed on contextual memory (see also^15^, ^21^). The sparing of spatial but not contextual memory retrieval after stress in the CSD task agrees with studies showing that flexible forms of memory are more vulnerable to the deleterious effects of stress as compared to stable ones.^51^ Spatial memory in CSD procedure evaluates a reference memory component. In contrast, “correct” responses depended on the retrieval of a unique internal context (floor) which is associated to a specific spatial location; thus, contextual memory could be an index of the flexible memory processes involved in declarative‐like memory in animals.^48^ A specific contribution of our study is to show that this improvement is observed on the retrieval phase of memory processes, and both in nonstress and stress conditions. Indeed, it has been reported that histamine may act differently on memory consolidation according to the emotional component of the task.^52^, ^53^, ^54^ The fact that a memory‐enhancing effect is observed in both nonstress and stress conditions in ciproxifan‐treated mice may be due to the fact that antagonists of H_3_ receptors have a wide range of effects on several neurotransmitters systems (acetylcholine, dopamine) involved at different levels in memory processes.

In the CSD task, in nonstress and stress conditions, both D1 and D2 are accurately and equally retrieved in ciproxifan‐treated mice, in contrast to nonstressed (D1 > D2) or stressed (D2 > D1) vehicle‐treated mice. Fos immnostaining showed that ciproxifan in nonstressed animals increased Fos immunoreactivity in the BLA and only a trend for prelimbic cortex and central amygdala which have previously been found to sustain memory retrieval of D2.^43^, ^44^ One would expect an increase of Fos levels in hippocampus of ciproxifan nonstressed animals because previous work showed a crucial role of hippocampus in D1 retrieval. This discrepancy may be due to the lack of sensitivity of Fos immunostaining and/or the behavior may be related to other brain area activity connected to the hippocampus (eg, enthorhinal cortex or medio‐dorsal thalamus). In contrast, in stress condition, there is no significant impact of ciproxifan on Fos immunoreactivity as compared to stressed vehicles which may explain the ciproxifan‐induced improvement of the retrieval of D1. One would expect increase of Fos immunostaining in dHPC in this group. This discrepancy may be due to the fact that stress already induced important Fos staining in these brain areas, preventing observation of an additional impact of ciproxifan on the number of immunopositive cells. It is also possible that the expression of Fos may change dynamically and distinctively over time after stress in ciproxifan‐treated mice as compared to stressed vehicles, preventing any observation of ciproxifan or stress 90 minutes after behavioral testing.

### 4.2 Acute stress, ciproxifan, anxiety, and corticosterone levels

Plasma corticosterone concentrations are increased after stress only in vehicle‐treated animals whereas ciproxifan blocked the stress‐induced plasma corticosterone increase. In contrast, studies have shown that the injection of histamine in the paraventricular nucleus of the hypothalamus activates the HPA axis*via* the release of CRH which in turn leads to massive release of corticosteroids.^55^ To explain this discrepancy, it could be hypothesized that ciproxifan (injected 35 minutes before blood sampling) could activate hippocampus and/or prefrontal cortex neurons^9^ that exert a negative feedback on the HPA axis activity, leading to dampened corticosterone levels.^56^ In the present experimental paradigm, ciproxifan did not increase corticosterone levels also in nonstressed animals probably via a masking effect because the behavioral task itself induces corticosterone release.^44^

It is known that the release of histamine induced anxiety‐like reactivity through the activation of H1 receptors.^39^, ^57^, ^58^, ^59^ Injection of H_3_ agonists induces anxiolytic‐like effects^40^, ^57^, ^58^ whereas antagonists induce dose‐dependent anxiogenic‐like effects.^57^ Thus, in the present study, one would have expected that the administration of ciproxifan would have modified emotional reactivity in the EPM task. Our results evidenced mitigated effects. Indeed, on the one hand, ciproxifan 3 mg/kg does not induce significant anxiogenic‐like effects in nonstressed mice; on the other hand, the deleterious effects of stress on the time spent in open‐arms were not observed in ciproxifan‐treated mice. Since the procognitive impact of ciproxifan in the CSD task is observed both in nonstress and stress conditions, this indicates that the 3 mg/kg procognitive dose of ciproxifan does not depend on its emotional or endocrinal effects, at least in our experimental conditions.

## 5 CONCLUSION

Ciproxifan 3 mg/kg enhanced contextual memory retrieval, both in stress and nonstress conditions and dampened emotional reactivity and glucocorticoid responses to an acute stress. Overall, this study emphasized the usefulness of H3 receptor antagonist to enhance cognitive functions both in stress and nonstress conditions.

## CONFLICT OF INTEREST

The authors declare that there is no conflict of interest.
